# Detection of serum soluble markers of immune activation in rheumatoid arthritis

**DOI:** 10.1155/S0962935196000373

**Published:** 1996-09

**Authors:** T. Kogure, T. Itoh, Y. Shimada, T. Shintani, H. Ochiai, K. Terasawa

**Affiliations:** 1 Department of Japanese Oriental Medicine Faculty of Medicine Toyama Medical and Pharmaceutical University 2630 Sugitani Toyama 930-01 Japan; 2 Department of Human Science Faculty of Medicine Toyama Medical and Pharmaceutical University 2630 Sugitani Toyama 930-01 Japan

## Abstract

The mutual correlation among soluble CD4 (sCD4), soluble CD8 (sCD8), and soluble CD23 (sCD23) has not yet been studied in patients with rheumatoid arthritis (RA), although previous studies have demonstrated that certain soluble markers of immune activation are elevated in RA. Thus, we examined this correlation based on the serum levels of sCD4, sCD8 and sCD23, and that of their levels with other serum markers such as immunoglobulin (Ig) subtypes (IgG, IgM and IgA), IgM-rheumatoid factor (IgM-RF) and C-reactive protein (CRP) in 25 RA patients, sCD4 was not elevated, whereas both sCD8 and sCD23 increased in RA patients compared with the healthy controls; a majority of RA patients, in particular, showed a high sCD23 level. The level of sCD23 showed a correlation with that of IgM-RF, but not with those of IgG, IgM, IgA and CRP. Importantly, a high level of sCD23 was not always accompartied with that of sCD8. The independent change between sCD23 and sCD8 levels was also observed in a one-year follow-up study of the two RA patients. These findings indicate that B cells might be generally activated in RA, whereas T-cell activation in variable in each patient with RA, suggesting that sCD23 is a more indicative marker for the immune status of RA patients than sCD8 from the clinical aspects.


Mediators of Inflammation 5, 262–265 (1996)

### Detection of serum soluble markers of immune activation in rheumatoid arthritis

T. Kogure[1,CA], T. Itoh[1], Y. Shimada[1], T. Shintani[1], H. Ochiai[2] and K. Terasawa[1]

Departments of [1]Japanese Oriental Medicine, and [2]Human Science, Faculty of Medicine, Toyama Medical and Pharmaceutical University, 2630 Sugitani, Toyama 930-01, Japan

[CA]Corresponding Author

**The mutual correlation among soluble CD4 (sCD4), soluble CD8 (sCD8), and soluble CD23 (sCD23) has not yet been studied in patients with rheumatoid arthritis (RA), although previous studies have demonstrated that certain soluble markers of immune activation are elevated in RA. Thus, we examined this correlation based on the serum levels of sCD4, sCD8 and sCD23, and that of their levels with other serum markers such as immunoglobulin (Ig) subtypes (IgG, IgM and IgA), IgM-rheumatoid factor (IgM-RF) and C-reactive protein (CRP) in 25 RA patients. sCD4 was not elevated, whereas both sCD8 and sCD23 increased in RA patients compared with the healthy controls; a majority of RA patients, in particular, showed a high sCD23 level. The level of sCD23 showed a correlation with that of IgM-RF, but not with those of IgG, IgM, IgA and CRP. Importantly, a high level of sCD23 was not always accompanied with that of sCD8. The independent change between sCD23 and sCD8 levels was also observed in a one-year follow-up study of the two RA patients. These findings indicate that B cells might be generally activated in RA, whereas T-cell activation in variable in each patient with RA, suggesting that sCD23 is a more indicative marker for the immune status of RA patients than sCD8 from the clinical aspects.**

**Key words:** Rheumatoid arthritis, Soluble CD4, Soluble CD8, Soluble CD23

### Introduction

Rheumatoid arthritis (RA) is a complex inflammatory disease of unknown cause and an autoimmune phenomenon is strongly suspected.[1,2] Previous studies have described the significant role of both T-cells and pro-inflammatory cytokines such as interleukin (IL)-1, IL-6 and tumour necrosis factor-α (TNF-α) in RA.[3–5] In addition, B cells in the rheumatoid synovium are believed to produce the rheumatoid factor (RF), of which controlled mechanisms are still unclear.[6] Similar to systemic lupus erythematosus, serum soluble CD8 (sCD8), soluble CD23 (sCD23) and soluble IL-2 receptor have been shown to become elevated in RA.[7–9] Because these molecules are recognized as being involved in T- and B-cell functions,[10,11] it is likely that the immune activation contributes in part to the pathogenesis of RA. However, a mutual correlation among the serum levels of these molecules has not yet been clarified in RA. Thus, we examined this issue based on the serum levels of sCD4, sCD8, and sCD23/(low-affinity receptor for the Fc portion of IgE), and their correlation with other serum markers such as immunoglobulin (Ig) subtypes (IgG, IgM and IgA), IgM-rheumatoid factor (IgM-RF) and C-reactive protein (CRP) in addition to erythrocyte segmentation rate (ESR) in 25 RA patients. Furthermore, a longitudinal comparison in the changes in the serum levels of certain soluble markers was performed.

### Materials and Methods

Twenty-five patients (19 females and six males) with flares of RA as defined by the revised criteria of the American College of Rheumatology[12] were included in this study. Characteristics of these patients were as follows: mean ± SD age 58.3 ± 13.5 years (range 22–76 years), mean ± SD disease duration 8.5 ± 6.4 years (range 1–25 years), mean ± SD ESR 81 ± 43 mm/h (range 35–115 μm/h), mean ± SD $CH_{50}$ 37.8 ± 7.1 mg/dl (range 26–53 mg/dl), anatomical stage 2.4 ± 1.2 and functional class 2.1 ± 0.9. All were receiving non-steroidal anti-inflammatory drugs (NSAIDs). Two were also taking bucillamine, three gold salts, and four prednisolone (PSL, 2.5–10 mg/day). None was receiving metho-

© 1996 Rapid Science Publishers

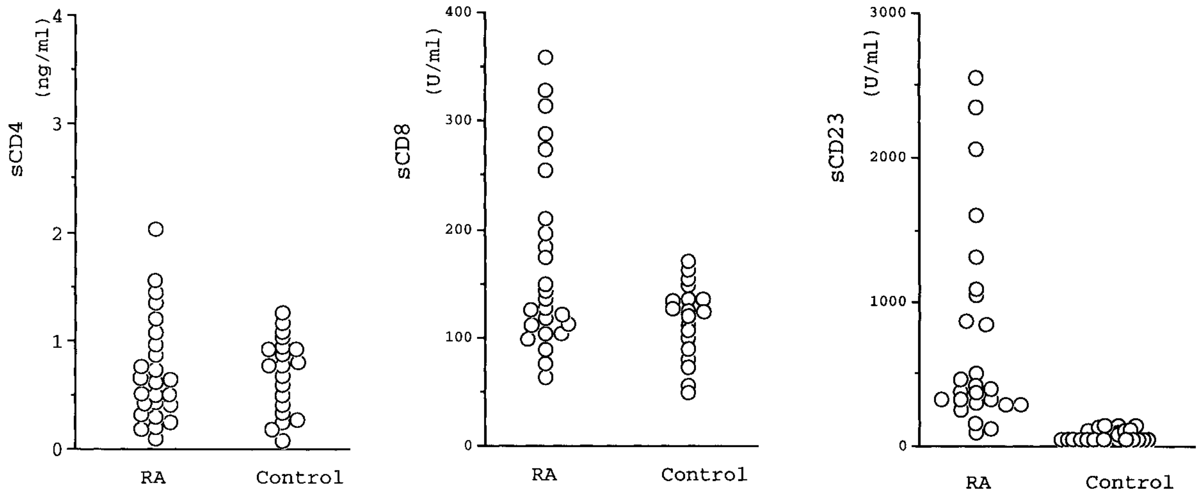

FIG. 1. Comparisons of serum sCD4, sCD8 and sCD23 levels between 25 RA patients and 20 healthy controls. The levels of sCD4 (left panel) were no different between RA and controls, whereas those of sCD23 (right panel) in RA patients were significantly elevated compared with controls. Although no significant difference was obtained in the mean value of sCD8 levels between RA and controls (middle panel), there was a tendency to show higher sCD8 levels in RA patients. The Mann–Whitney *U*-test was performed using the StatView-J 4.02 program (Abacus Concept, CA, USA).

trexate. As a control, sera from 20 healthy and age-matched volunteers (52.3 ± 15.9 years) were used. sCD4, sCD8 and sCD23 levels were determined simultaneously in all serum samples as described below.

Serum levels of the soluble markers were measured by antibody sandwich enzyme-linked immunosorbent assay (ELISA) using commercial kits (sCD8 and sCD23: T-cell Diagnostics, Inc., Cambridge, MA; sCD4: Boehringer Mannheim Biochemica, Germany). These levels were expressed as U/ml except for sCD4 which was expressed as ng/ml based on the standard curve prepared simultaneously using standard molecules provided in each kit. The detection limits were 0.25 ng/ml, 50 U/ml and 15 U/ml for sCD4, sCD8 and sCD23, respectively. Serum samples were stored at –20°C until use.

Table 1. Correlation of sCD23 with several serological markers

| sCD23 versus | Probability | Spearman's rank correlation coefficient |
|---|---|---|
| IgG | 0.976 | 0.008 |
| IgM | 0.243 | 0.290 |
| IgA | 0.363 | 0.139 |
| CRP* | 0.608 | 0.089 |
| ESR** | 0.108 | 0.570 |
| IgM-RF | 0.005 | 0.505 |

*CRP: C-reactive protein, **ESR: erythrocyte segmentation rate.

All data were collected in a computer database and analysed using Spearman's rank correlation in the StatView-J 4.02 program.

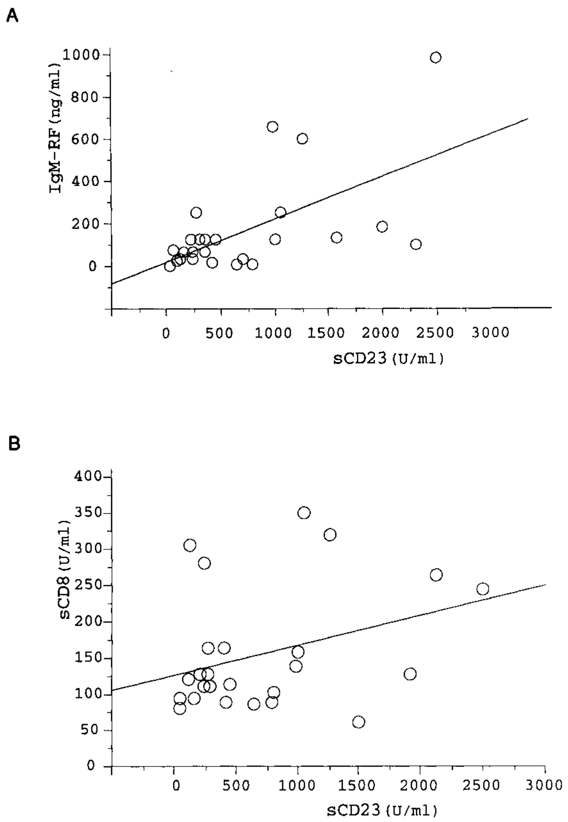

FIG. 2. Correlation of sCD23 with IgM-RF, or with sCD8 in 25 RA patients. Correlation between sCD23 and IgM-RF was analysed (A) as described in the legend for Fig. 1. The data were summarized as follows: Spearman rank correlation coefficient: $r = 0.505$, $p = 0.0049$; linear regression coefficient: $r = 0.614$, $p = 0.0002$; $y = 0.2x + 20.053$. Alternatively, the same analysis was carried out between sCD23 and sCD8 (B). The data were summarized as follows: Spearman's rank correlation coefficient: $r = 0.261$, $p = 0.200$; linear regression coefficient: $r = 0.326$, $p = 0.112$. As a result, a significant correlation was obtained in the former analysis but not in the latter one.

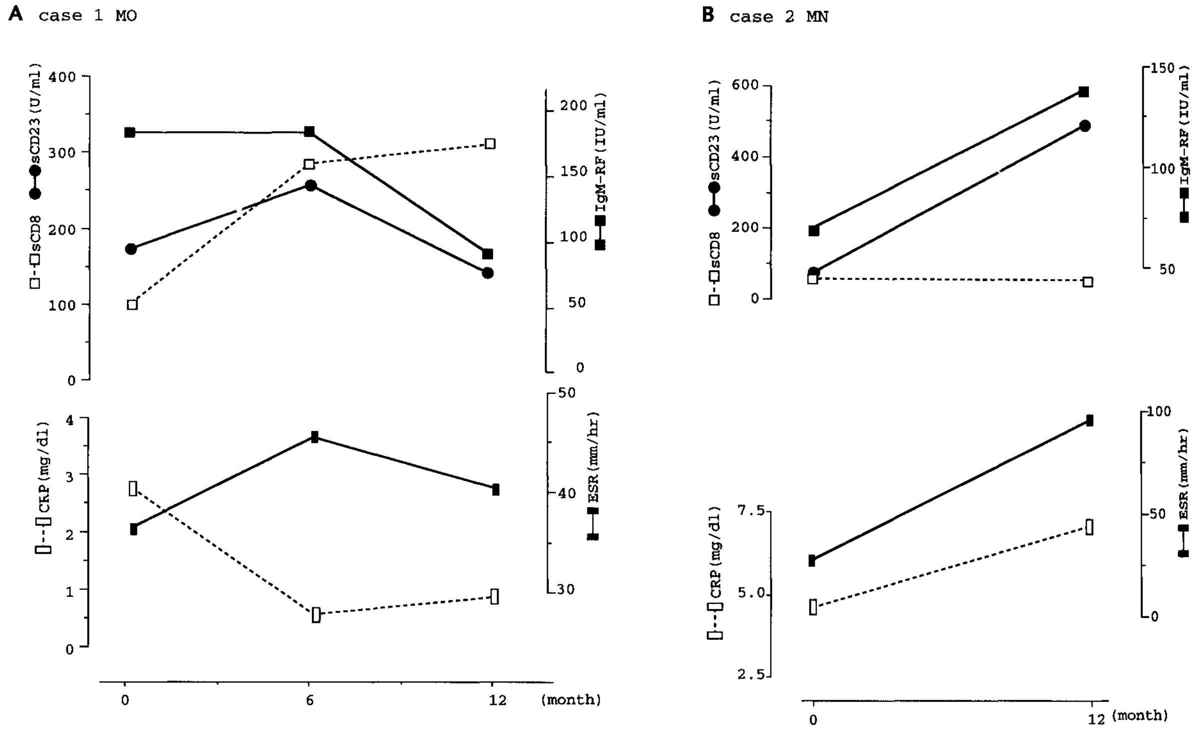

FIG. 3. The time-related changes in the serum sCD23 level and other parameters (sCD8, IgM-RF, CRP and ESR) in two RA patients during one year. Study periods were from June 1994 to July 1995 for Case 1 (M.O., female, aged 70 years) with RA since 1968 (A), and from July 1994 to June 1995 for Case 2 (M.N., female, aged 59 years) with RA since 1993 (B). In both cases, levels of sCD23 and sCD8 were measured simultaneously in the sera which were stored at -20°C.

### Results and Discussion

As shown in Fig. 1, the serum level of sCD4 showed no significant difference between RA patients and healthy controls, but that of sCD23 was significantly higher in all RA patients compared with controls. Concerning sCD8, these values were distributed in a wider range with a tendency that the majority of RA patients showed higher ones which were, as a whole, statistically no different from the controls. When the correlation of the level of sCD23 with that of each Ig subtype, CRP, or ESR was examined (Table 1), no correlation was obtained between sCD23 and each Ig subtype, or between sCD23 and CRP. However, an insignificant but considerable correlation was shown between sCD23 and ESR. Considering the fact that CRP is well known as a more acute phase reactant than ESR, this finding suggests that the serum level of sCD23 might be taken as a parameter reflecting the immune activation in the chronic phase rather than the acute phase. On the other hand, the correlation between serum levels of sCD23 and IgM-RF was statistically significant (Fig. 2A). These data confirm that sCD23 might play an important role on the specific production of IgM from RF-committed B cells.

A recent study has revealed that the sCD23 molecule is not only a soluble activation marker, but also a proinflammatory cytokine achieving an important role in the control of the immune response including T-cell response.[13] Thus, we further investigated the relationship between sCD23 and sCD8. As shown in Fig. 2, a correlation was not shown between these two molecules, indicating that B cells might be activated generally in RA, whereas the degree of T-cell activation varies patient by patient in RA. Thus, it is likely that a higher level of sCD23 in RA patients does not always lead to the T-cell activation. The analysis of the relation between sCD23 and sCD4 was not performed, because the normal level of sCD4 was observed in RA (see Fig. 1).

We further performed a longitudinal study on the changes in the serum sCD23, sCD8, and IgM-RF levels in addition to classical inflammation parameters (CRP and ESR) in two patients during one year. The results are shown in Fig. 3. In both cases, the serum levels of sCD23 were changing almost in parallel with that of IgM-RF.

Comparing both cases, a changing pattern of sCD23 was correlated more closely to that of ESR than CRP. In contrast, time-related change of the serum level of sCD8 was also independent from that of sCD23 in this study as shown in the cross-sectional study described above. These observations might permit speculation that sCD23, but not sCD8, is taken as a more indicative marker reflecting the immune status of RA patients either in cross-section or longitudinal study.

### References

1. Zvaifler NJ. Immunopathology of joint inflammation in rheumatoid arthritis. *Adv Immunol* 1973; **16:** 265–336.
2. Firestein GS, Tsai V, Zvaifler NJ. Cellular immunity in the joints of patients with rheumatoid arthritis and other forms of chronic synovitis. *Clin Rheum Dis* 1987; **13:** 191–213.
3. Panayi GS, Lanchbury JS, Kingsley GH. The importance of the T cell in initiating and maintaining the chronic synovitis of rheumatoid arthritis. *Arthritis Rheum* 1992; **35:** 729–735.
4. Ruschen S, Stellberg W, Warnatz H. Kinetics of cytokine secretion by mononuclear cells of the blood from rheumatoid arthritis patients are different from those of healthy controls. *Clin Exp Immunol* 1992; **89:** 32–37.
5. Westacott CI, Whicher JT, Barnes IC, Thompson D, Swan AJ, Dieppe PA. Synovial fluid concentration of five different cytokines in rheumatic disease. *Ann Rheum Dis* 1990; **49:** 676–681.
6. Vaughan JH, Chihara T, Moore TL, Robbins DL, Tanimoto K, Johnson JS, McMillan R. Rheumatoid factor-producing cells detected by direct haemolytic plaque assay. *J Clin Invest* 1976; **58:** 933–941.
7. Zielinski CC, Pesau B, Muller CH. Soluble interleukin-2 receptor and soluble CD8 antigen in active rheumatoid arthritis. *Clin Immunol Immunopathol* 1990; **57:** 74–82.
8. Chomarat P, Briolay J, Banchereau J, Miossec P. Increased production of soluble CD23 in rheumatoid arthritis, and its regulation by interleukin-4. *Arthritis Rheum* 1993; **36:** 234–242.
9. Symons JA, Wood NC, Di Giovine FS, Duff GW. Soluble CD8 in patients with rheumatic diseases. *Clin Exp Immunol* 1990; **80:** 354–359.
10. Delespesse G, Suter U, Mossalayi D, Bettler B, Sarfati M, Hofstetter H, Kilcherr E, Debre P, Dalloul A. Expression, structure and function of the CD23 antigen. *Adv Immunol* 1991; **49:** 149–191.
11. Gordon J. CD23 and B cell activation. *Clin Exp Allergy* 1992; **22:** 199–204.
12. Arnett FC, Edworthy SM, Bloch DA, McShane DJ, Fries JF, Cooper NS, Healey LA, Kaplan SR, Liang MH, Luthra HS, Medsger TA Jr, Mitchell DM, Neustadt DH, Pinals RS, Schaller JG, Sharp JT, Wilder RL, Hunder GG. The American Rheumatism Association 1987 revised criteria for the classification of rheumatoid arthritis. *Arthritis Rheum* 1988; **31:** 315–324.
13. Armant M, Ishihara H, Rubio M, Delespesse G, Sarfati M. Regulation of cytokine production by soluble CD23: costimulation of interferon $\gamma$ secretion and triggering of tumor necrosis factor $\alpha$ release. *J Exp Med* 1994; **180:** 1005–1011.

ACKNOWLEDGEMENTS. The authors wish to thank Miss Terue Kataoka for excellent assistance. This work was supported in part by a grant for the research of Oriental Medicine from the Tokyo Metropolitan Government.

**Received 24 April 1996; accepted 14 May 1996**
